# A Practical Treatise on the Diseases of Children

**Published:** 1845-11

**Authors:** 


*A Practical Treatise on the Diseases of Children.* By JAMES STEWART, M. D., A. M., Fellow of the College of Physicians and Surgeons of New York, &c. &c. *Third edition, carefully revised and enlarged.* 8vo. pp. 544. Harper and Brothers, New York, 1845.

When we read the title page of this publication, we expected, as we presume any one else would, that it was something different from the second edition—that it had really been "*carefully revised and enlarged*," and we turned over its pages with some anxiety to see wherein it had been "*revised*," and to what extent and in what manner it had been "*enlarged*." Alas! we could find no evidence of either. The first thing that struck our eye was that it contained precisely the same number of pages as the second edition, and that the matter of the last page terminated just in the same manner and place in each—that the *Index* in each edition commenced on page 542, and ended on page 544—that the matter or text of the volume, in both cases, not only terminated in each on page 541, but in precisely the same part of the page, and in both cases with a "*Note to Vaccination*," consisting of the same number of lines, and containing precisely the same words, and exactly the same typographical peculiarities. On comparing different parts of the two editions, we found the same identity throughout. We recollected then that the second edition was stereotyped, and we saw at once that the present, or so-called *third edition*, was nothing more nor less than the striking off of an additional number of copies from the same unaltered plates, with no revision, and no enlargement whatever! The only changes discernible are in substituting *third* for *second* edition, the names of "Harper and Brothers" as publishers, for the Messrs. Langley, and in the substitution of 1845 for the year in which the former edition was published. On the back of the title page, too, the annunciation that the work was stereotyped, contained in the former edition, is omitted in this! Why is this? The treatise is a good one, and needs no meretricious means of support; we must presume therefore that the alteration made in the title page was done hastily, and without thinking of the inconsistency it involved—otherwise, it would not comport with the high character of the eminent publishing house from whence it proceeds.

